# Graph Neural Networks in Cancer and Oncology Research: Emerging and Future Trends

**DOI:** 10.3390/cancers15245858

**Published:** 2023-12-15

**Authors:** Grigoriy Gogoshin, Andrei S. Rodin

**Affiliations:** Department of Computational and Quantitative Medicine, Beckman Research Institute, and Diabetes and Metabolism Research Institute, City of Hope National Medical Center, 1500 East Duarte Road, Duarte, CA 91010, USA

**Keywords:** graph neural network, GNN, deep learning, cancer, oncology, graphical model, Bayesian network

## Abstract

**Simple Summary:**

Graph Neural Networks are emerging as a powerful tool for structured data analysis, and predictive modeling in massive multimodal datasets. In this review, we survey recent applications of graph neural networks in the setting of cancer and oncology research. We identify currently predominant research areas, and compare graph neural networks with non-graph deep learning methods as well as probabilistic graphical models. We conclude by highlighting emerging trends and pressing challenges, such as developing independent and comprehensive benchmarking frameworks. This review is aimed at cancer and oncology researchers, clinicians and physician-scientists who are interested in applying graph-centered secondary data analysis methods to structured multimodal data.

**Abstract:**

Next-generation cancer and oncology research needs to take full advantage of the multimodal structured, or graph, information, with the graph data types ranging from molecular structures to spatially resolved imaging and digital pathology, biological networks, and knowledge graphs. Graph Neural Networks (GNNs) efficiently combine the graph structure representations with the high predictive performance of deep learning, especially on large multimodal datasets. In this review article, we survey the landscape of recent (2020–present) GNN applications in the context of cancer and oncology research, and delineate six currently predominant research areas. We then identify the most promising directions for future research. We compare GNNs with graphical models and “non-structured” deep learning, and devise guidelines for cancer and oncology researchers or physician-scientists, asking the question of whether they should adopt the GNN methodology in their research pipelines.

## 1. Introduction

Next-generation cancer research is increasingly moving towards the full integration of big data, machine learning (ML) approaches (including deep learning, DL), and computational systems biology methods, with the latter concentrating on constructing, curating, interpreting, and validating various multimodal biological network models [1]. One of the primary challenges in ongoing and future computational cancer and oncology research is the appropriate selection and integration of the many complementary yet overlapping high-dimensional multiscale analysis and modeling methods, usually vaguely gathered together under the umbrella of “AI”. A practitioner, be it a cancer researcher, a clinician, or a physician-scientist, is often overwhelmed by the sheer repertoire of the AI/ML/network-centered analysis and modeling methodology at their disposal. Moreover, this repertoire is growing daily, and, while presenting an enormous opportunity, such a methodological cornucopia is also a challenge, requiring a clear understanding of the scope, applicability, and limitations of the computational algorithms and tools. This is exacerbated by the frequently equivocal terminology, reflecting parallel research progress in computer science and AI, multivariable statistics, and graph theory and network science.

One of the most interesting, and promising, recent developments in DL has been the advent of graph neural networks (GNNs). Although combining graph structures with DL was codified as early as 2005–2009 [2,3,4], GNNs did not attract broad attention in the bioinformatics, computational biology, and computational chemistry communities until 2019–2020 (following the general explosion of DL, and DL applications in life sciences). A recent (October 2023) MEDLINE/PubMed search query (“graph neural network” OR “graph neural networks”) AND (“oncology” OR “cancer”) generated 151 results (4 in 2020, 26 in 2021, 59 in 2022, 67 in 2023), suggesting an emerging trend. Dissecting this trend is the principal goal of this review.

The application of GNNs in cancer research and oncology holds an immediate appeal because GNNs are intuitively understood as a synthesis of graph structures (naturally representing, for example, multiscale biological networks, or molecular structures, or knowledge graphs) and powerful DL approaches; however, there is a certain amount of confusion about the relationship between GNNs and other, more “conventional” in the life sciences context, network-centered methods—such as co-expression networks, gene regulatory networks, network enrichment analysis, Bayesian networks, Markov networks, etc. This confusion leads to the often-asked question: how are GNNs different from the other network methods, and should they supersede the latter in a prototypical cancer researcher’s computational systems biology toolkit? Concurrently, another question arises: what is the added value that GNNs can bring to a cancer researcher, compared to the other leading-edge DL techniques that can accommodate non-homogenous, structured data? These two inquiries provided the original impetus for the present review.

In this review, we aim to specifically address the following questions:

1. What are the emerging trends in the application of GNN methodology to cancer and oncology research? Are there any fields and sub-fields in which the GNNs are poised to predominate? 2. Consequently, should cancer and oncology researchers consider GNNs in addition to, or instead of, more established DL approaches? Furthermore, if yes, under which scenarios and circumstances? What are the added benefits, if any? 3. Likewise, should the cancer and oncology researcher community reevaluate more established non-DL network modeling approaches and consider augmenting or replacing them with GNNs? The structure of the rest of this review is as follows: first, we introduce the GNN methodology fundamentals, and compare them to graphical models. Then, we survey the recent trends in GNN applications in cancer research and oncology and highlight several fields in which the GNN approach appears to be the most efficacious. Finally, we compare and contrast GNNs with non-graph DL and non-DL network-centered methods, and conclude by identifying promising future trends and research directions.

It should be emphasized that this review is intentionally focused in its scope, namely on the **practical applications of GNNs in the context of cancer and oncology research.** As such, this review is aimed at practitioners asking a very specific question: should they incorporate the novel GNN methodology in their cancer and oncology research pipelines? To gain a broad and complementary perspective on AI/DL in cancer and oncology research beyond the scope of this communication, we refer the reader to the recent reviews on explainable AI in oncology [5], AI in lung cancer [6], interpretable DL in oncology [7], DL in imaging/cancer diagnosis [8], GNNs in imaging/histopathology [9,10,11], GNNs in bioinformatics [12], AI in cancer multiomics [1], DL in drug response prediction in cancer cell lines [13], and DL in biological networks [14].

## 2. GNN Fundamentals

A graph, or network, is a data structure with high expressive power that consists of nodes and edges (reflecting the relationships between nodes). In life sciences, such networks can be very high-dimensional (-omics data) or very multimodal (from molecular data to clinical data to communities and social networks) or both. Merging graph representation with DL can be achieved by adapting DL’s inputs and outputs to non-Euclidian data, wherein various graph features (nodes, edges, sub-networks, or whole graphs) are transformed into low-dimensional vectors in the process of graph embedding. However, contextual topological information might be lost in encoding/embedding; a more “generalist” GNN approach iteratively updates node states in the graph via message passing between the nodes in a manner similar to DL but with a local topology (i.e., a complement of neighboring nodes) taken into account. A variety of GNN models have been proposed with some of the more prominent ones being spectral-based and spatial-based GCNs (Graph Convolution Networks) [15,16], Graph RNNs (Graph Recurrent Neural Networks) [17], GATs (Graph ATtention networks) [18], and GAEs (Graph AutoEncoders) [19]. We refer the reader to the excellent recent reviews on GNNs [12,20,21] for technical details and classification of different GNN approaches and implementations; here, we will only note that, similar to non-DL network modeling methods, graph topology can be pre-set (e.g., representing a molecular structure, a spatially resolved image, or expert knowledge in the domain), or can be learned from data, via model selection. Likewise, the learning tasks/outputs of GNNs are similar to those in the non-DL network analyses: node-level (value of a node of interest), graph-level (property of the entire graph), and edge-level (edge detection) predictions, with the latter generalizing to the aforementioned learning of the (sparse) graph topologies from data. In summary, GNNs promise to combine the high expressivity and inherent interpretability of graph structures (and their natural congruence with many life science research and clinical data types) with the predictive/learning power of DL.

## 3. GNNs and Graphical Models

GNNs are superficially similar to graphical models, in that both perform learning over graph structures. Bayesian networks (BNs), or probabilistic directed acyclic graphs (DAGs) learned from the data, are arguably the most popular graphical models in life science applications. BNs can incorporate both data-driven learning and existing knowledge, and allow for probabilistic reasoning and propagation over the DAGs. A major feature of BNs is that they filter out superficial (transitive, non-direct) dependencies, thus arriving at sparse DAGs suggesting directional causalities [22,23,24]. A question is often asked: what are the principal differences between BNs and GNNs, especially from the life sciences application perspective? Here, we compare the underlying fundamentals of a GNN (specifically, a GCN) and a BN.

In many cases, a graph is simply an abstraction defined over another model that can be written algebraically. This is the case for graphical models, such as BNs, where a probabilistic model is the basis for its graphical representation. Additional constraints of directionality and acyclicity are imposed on the graph representation by the underlying probabilistic model (hence, a DAG), although, generally, not in a unique way. In addition to the usual pairwise interactions, BNs are capable of modeling probabilistic dependencies of very high-order and almost arbitrary depth. This is one of the reasons that BNs are often perceived to stand in correspondence with causal structures. However, while causal inference is certainly possible with BNs in some circumstances, the notion of causation is usually much narrower than probabilistic dependency. BNs are well-equipped for probabilistic reasoning in contexts with a high degree of uncertainty where little a priori information about the nature of the interaction in question is available. Although BNs do not expect temporal ordering required for causal inference, they can readily accept causal constraints.

**Figure 1:**
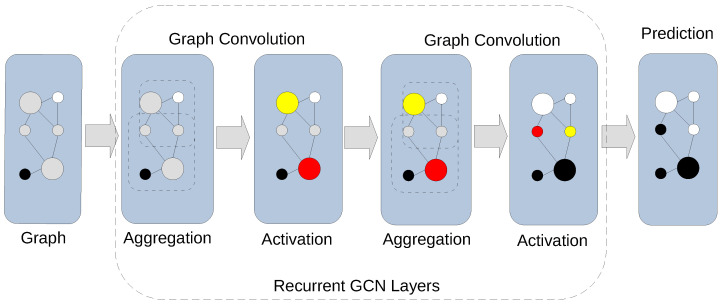
Graph convolution in a GCN. Recurrent graph convolution layers of a GCN contain aggregation and activation stages. An aggregation stage combines feature information from its neighborhood. An activation stage applies a non-linear activation function to the result of the aggregation stage. Recursive application of convolution layers agglomerates information across distant neighborhoods enabling prediction of class labels for unlabeled nodes.

**Figure 2:**
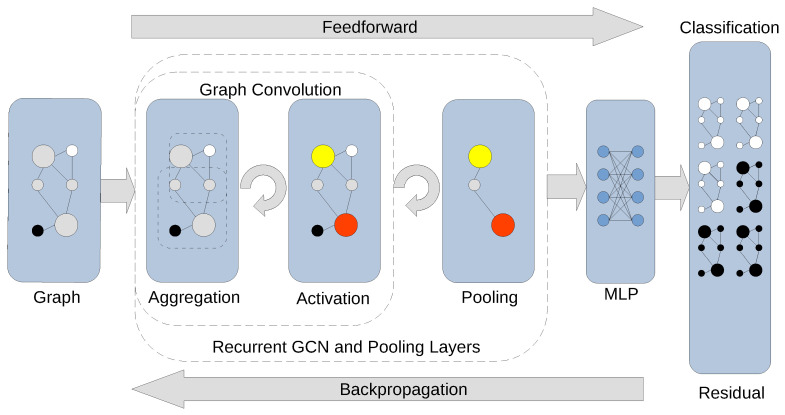
Graph classification via recurrent GCN layers combined with pooling layers. A convolutional layer stacked with a pooling layer builds a more abstract coarsened graph representation of the original graph. The final pooling layer encodes the graph into its most general representation, followed by a graph classification in the fully connected layer (MLP, Multilayer Perceptron) with an appropriate activation function. The output of the MLP layer constitutes class label assignment, which places the graph into its destination category. The residual of checking label assignments against the training data can be backpropagated through the model to update the weights in modifiable layers.

While both rely on graph representations, GNNs are quite different from BNs. Typically, a GNN relies on information diffusion techniques, e.g., graph convolution in the case of a GCN (Figure 1), to accomplish a graph-relevant predictive task such as the classification of nodes. In the simplest configuration, a feedforward GCN, for example, maps an aspect of a graph to a numerical scale of an appropriate dimension. A GCN with backpropagation (Figure 2) can approximate the mapping between certain aspects of a graph and its class assignment from examples. From this perspective, a GNN is one of many generic approximation methods that establish a relationship between a graphical model and its implications.

Conversely, a BN is a dependency model, or, more precisely, a way to specify the probabilistic model for essential dependencies between various observables. A properly defined BN contains all the information necessary to reconstruct the associated joint probability distribution and, therefore, makes node-wise prediction a matter of probabilistic inference. Estimation of BN structure and parameters from observations constitutes an inverse problem that can be approached in a variety of ways. Once a BN model is obtained, the information contained therein can be interpreted directly, without the aid of additional methodological devices, and utilized for probabilistic inference as well as for construction of classifiers, predictors, and other tools for a particular knowledge or problem domain.

GNNs and BNs serve largely complimentary purposes with little (but not insignificant) instrumental overlap. Under some assumptions, a BN can be aided in its specification by a GNN [25] in a way similar to classical parameter estimation methods [26]. However, once a BN is completely specified, it is a more efficient stand-alone tool for any kind of inference task over the problem domain, including prediction and classification. More importantly, it makes the accumulated problem domain knowledge explicit and directly interpretable, which enables the design of highly efficient problem-specific methods. In this, a multiscale BN stands in contrast to the largely “black box” nature of a DL model, even one containing GNN components.

An analogy that makes this difference (explicit domain knowledge and direct interpretability vs. “black box” with or without ex post facto explainability) clear is the approximation of a signal, or image, via expansion into a spectral basis as opposed to conventional interpolation. Here, the basis may have domain-specific meaning, e.g., the trigonometric basis in Fourier series representation. A more generic spline interpolation may perform equally well or even better than spectral expansion, but it leaves out the explicit interplay of parameters that occurs in the frequency domain along with the possibility of spectral manipulation. Thus, spectral methods offer a clear interpretational advantage. A notable example is spectral CT (Computed Tomography) [27], where image enhancement relies on the frequency-dependent or energy-dependent attenuation of different tissues. Spectral information not only drastically enhances tissue differentiation, but also carries domain-specific content that aids in identifying specific types of material [28], underscoring the practical benefits of increased interpretability.

In summary, the application of BNs accentuates inference over the problem domain, knowledge representation, and construction of narratives and hypotheses. Conversely, GNNs are well-equipped to deal with generic approximation tasks in which the way this approximation can be achieved and how informative it must be are not the primary concerns.

We will discuss the practical considerations behind the choice between GNNs and graphical models below in Section 5.

## 4. GNN Applications in Cancer Research and Oncology

In surveying the field, two major themes emerge: interpretability and multimodality. The graph structure representation underpinning GNNs is inherently interpretable in contrast to the ex post facto explainability in DL (aka explainable AI, or XAI) [29], and can naturally combine different modalities/data types within a single analysis framework. In addition, and on different abstraction levels, graph representation is a natural fit with the molecular structures and the image data types. These three advantages of GNNs—(i) inherent interpretability, or intelligibility (providing a potential pathway to causal discovery); (ii) combining different modalities/data types/scales; and (iii) natural representation of molecular structures and images—led to the recent and ongoing (2019–2023) cancer and oncology research GNN-centered work. After manually curating and augmenting 151 publications resulting from the MEDLINE/PubMed search (see Section 1 above), we identified 90 original use cases, representative of the current state-of-the-art research landscape, that concentrate predominantly in the following (partially overlapping) six major areas of activity:

1. Using multimodal data (including imaging, histopathology, and digital pathology) for cancer diagnosis, prognosis, survival, and therapy response prediction;
2. Cancer classification, subtyping, and grading;
3. Granular spatial approaches (including transcriptomics and proteomics);
4. Cancer drug selection, repurposing, and profiling; prediction of cancer drug interactions and combinations, response, and resistance.;
5. Synthetic lethality prediction;
6. Prediction of ncRNA (miRNA, piRNA, lncRNA) and circRNA–cancer associations.

**Figure 3:**
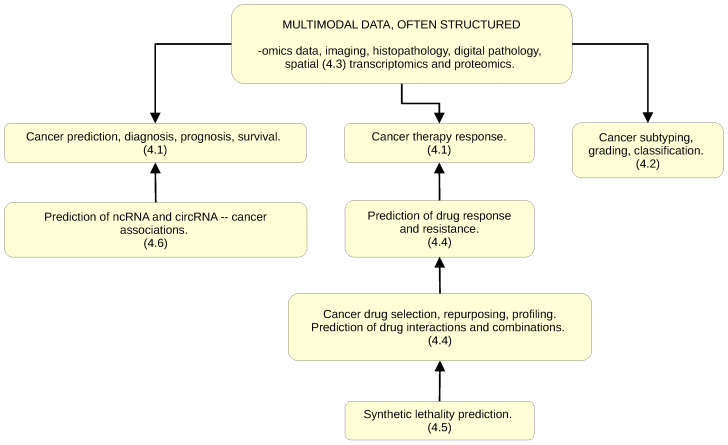
Applications of GNNs in cancer and oncology research: major areas of activity. Indices (4.1–4.6) refer to Section 4.1, Section 4.2, Section 4.3, Section 4.4, Section 4.5, Section 4.6 below.

Before proceeding to the description and analysis of use cases (Figure 3, Section 4.1, Section 4.2, Section 4.3, Section 4.4, Section 4.5, Section 4.6, Section 4.7 below), it should be emphasized that, although intrinsic interpretability is a significant pragmatic consideration, the actual performance (in prediction/classification, typically summarily assessed in this setting via AUC-ROC, Area Under the Receiver Operating Characteristic Curve, analysis) of GNN-based approaches often proves superior to conventional DL approaches as well. This can be explained by the higher congruence of the graph structure representations with the mechanistic/causal structure of the domain, thus making the inputs’ encoding/embedding less prone to information loss (which occurs due to the data type conversions and contextual information loss). In addition, just as with DL in general, GNNs tend to perform better than “classic” ML on the large datasets.

While there are few, if any, independent and comprehensive cross-benchmarking studies comparing GNNs with non-graph DL and non-DL ML in the cancer and oncology research settings, there is a growing recent effort towards developing principled performance benchmark frameworks attuned to GNNs [30,31] in the broader context. In parallel, there is an ongoing effort to independently cross-benchmark GNNs in various areas of computational chemistry [32,33] The overall preliminary conclusion is that GNNs tend to perform as well as, or oftentimes better than, non-graph DL/ML on the predominantly graph-level (and occasionally node-level and edge-level) tasks if the input data is structured.

### 4.1. Using Multimodal Data (Including Imaging, Histopathology, and Digital Pathology) for Cancer Diagnosis, Prognosis, Survival, and Therapy Response Prediction

Early work in this area focused on using GCNs [34] and GATs [35] to predict cancer phenotypes [35] and survival [34] from multimodal genetic, genomic, and clinical data, such as available in The Cancer Genome Atlas (TCGA). These approaches showed incrementally but significantly superior performance on prediction tasks compared to conventional ML and DL methods. Gao et al. [36] and Kim [37] extended the basic framework to model inter-patient groupings, “patient similarity networks”, likewise achieving performance improvements in survival prediction on different cancer datasets. Liang et al. [38] incorporated topological features of pathway representation of the transcriptomic data into the cancer survival prediction models for four cancers, taking advantage of the natural pathway–graph structure mapping. Again, prediction performance was superior to that of conventional ML/DL, with an added value of most predictive pathways’ delineation.

Subsequent work gradually incorporated imaging, histopathology, and digital pathology data—modalities that are particularly amenable to the graph structure representations. Lian et al. [39] used GCN with CT imaging data to predict lung cancer survival, achieving superior generalization prediction accuracy. Lee et al. [40] used GAT with digital pathology data (whole slide images, WSIs) to dissect features of the heterogeneous tumor microenvironment and predict the prognosis for four different types of cancer; importantly, the resulting models were interpretable at the contextual features level, underscoring the conceptual advantages of GNNs over typical “black box” DL predictors. Lian et al. [41] combined imaging data with clinical modalities in a transformer–GNN model to achieve superior risk and survival prediction performance for the early stage non-small cell lung carcinoma. Wang et al. [42] integrated multiplexed immunohistochemistry images into GNN models, thus enabling precise (binary and ternary classes) survival prediction in gastric cancer, with high multivariate prediction accuracy. Combining histopathology with computed topological features in a GNN model led to a significant improvement (0.956 average AUC compared to 0.911 average AUC for non-graph attention-based DL) in the accuracy of differential diagnosis of pancreatic ductal adenocarcinoma, a notoriously lethal human cancer [43].

Ding et al. [44] integrated CT data and clinical factors in a GAT model to achieve lymph node metastasis prediction superior (0.872 AUC) to that of single-modality approaches (0.797–0.853 AUC). Likewise, Hu et al. [45] developed a GNN forest model for highly accurate lymph node metastasis prediction that combined CT imaging, clinical features, and expert knowledge. An interesting aspect of this latter study was the medical experts’ involvement in the intermediate analysis stage (construction of the imaging-clinical super-graph). The WSI-data-based GNN model for the abnormal (non-neoplastic and neoplastic) endoscopic large bowel biopsy diagnosis developed by Graham et al. [46] also included an iterative interaction between a human expert (pathologist) and purely data-driven decision-making. To paraphrase a common witticism, the future might lie not in AI replacing human experts, but rather in human experts augmented by AI outperforming those without.

Recently, more complex, specialized GNN architectures have been proposed in the context of cancer prognosis/survival prediction. Fu et al. [47] developed a two-module GNN model combining clinical features with highly multiplexed imaging data that improved survival prediction on public breast cancer datasets. Zhu et al. [48] incorporated geometric features into sparse DL architectures, thus devising “geometric” GNNs that demonstrated high survival prediction accuracy on 11 different cancer types based on multiomic data. Zhang et al. [49] proposed a complex feature generation/GNN architecture to improve cancer prognosis prediction by combining multiomic data and molecular interactions in biological networks. Li et al. [50] developed a convolutional neural network (CNN)–GNN architecture for multimodal diagnosis of lung adenocarcinoma that used fused feature vectors to localize information transmission patterns, thus improving explainability. Notably, the four above studies demonstrate how a more complex, customized GNN/DL architecture can outperform “out-of-the-box” GNN solutions, signifying an emerging trend and suggesting that GNN applications in cancer and oncology research have reached maturity. Another sign of this growing maturity is an increasing emphasis on inferring causality, which naturally dovetails with the GNN paradigm. For example, Li et al. [51] set out to disentangle causative and non-causative tumor features in the context of GNNs using CT imaging data for early diagnosis of pancreatic cancer. Yet another direction for GNN refinement is the training mode. Azher et al. [52] compared different pretraining strategies for multimodal (methylation, expression, histopathology) GNN-based cancer prognostication and concluded that appropriate pretraining strategies might be more important than innovations in model architectures for highly accurate prediction.

Prediction of cancer therapy response is another task that is well-suited for multimodal GNN application. Wang et al. [53] utilized a CNN–GNN model to predict response to neoadjuvant therapy in rectal cancer using digital pathology data (WSIs), achieving high generalization prediction accuracy. Integrating multiple prior knowledge networks (gene–gene interaction graphs) in a GNN model enabled Zhao et al. [54] to attain superior prediction accuracy (up to 0.85 AUC compared to 0.62–0.74 AUC for single modalities) for immunotherapy (immune checkpoint inhibitor) response across different cancer types. The latter study showcases GNNs’ ability to seamlessly incorporate prior knowledge (which is often hard-coded in a graph structure form).

In summary, the application of GNNs to cancer diagnosis, prognosis, survival, and therapy response prediction is now a mature field. The emphasis is shifting from the straightforward implementations to various refinements of GNN architectures (and multistack DL architectures containing GNN modules) and training regimes, specific to the cancer-related predictive features and modalities. Two additional emerging trends are: (i) inferring causality, and (ii) an iterative human expert–AI predictor dialog, with both drawing on the inherent interpretability of the GNN representation.

### 4.2. Cancer Classification, Subtyping, and Grading

Methodologically, these applications overlap with Section 4.1 above, and have evolved in parallel. Early work [55] laid out the foundations for the typical analysis pipeline: use a GCN in conjunction with high-resolution (revealing a microarchitecture) histology images to construct large cell-level graphs incorporating multilevel features for grading of colorectal cancer. Likewise, Lu et al. [56] combined high-resolution digital pathology data (WSIs) with a customized GNN architecture to predict HER2 status in breast cancer; thus moving from the “patch” (wherein the large size WSI is subdivided into small tiles, or patches, for parallel DL applications with subsequent pooling) to the “entire WSI” level. This allowed the patch-level analysis shortcomings of limited visual context and absence of labeled granular data to be overcome. Pati et al. [57] developed a multiscale, hierarchical “cell-to-tissue” GNN for histopathological image classification and comprehensively surveyed early (2019–2021) work on graph structures (including GNN approaches) in digital pathology. In a similar vein, Wang et al. [58] added another hierarchical level—“cell communities” and their topological features—to the GNN analysis framework; emphasis on the topological data analysis led to a higher performance on pathology image classification, and disease grading tasks with multiple cancer types. Going one step further, Abbas et al. [59] developed a multicell type and multilevel graph aggregation architecture that takes into account both local and global cell–cell interactions and outperforms both CNNs and GNNs on cancer grading of digital pathology images.

Zhang et al. [60] used a different modality, distance-based features extracted from limited CT samples, to develop a GNN predictor for pancreatic cystic neoplasm classification; the dataflow followed a by-now established scheme—use a CNN to generate features and a GNN to complete the classification. Similarly, Ravinder et al. [61] combined CNN and GNN to improve brain tumor type classification using MRI images. Whereas, Ma et al. [62] proposed a dual GCN–GAT architecture for MRI brain tumor segmentation. Yin et al. [63] used yet another modality, multiomics, to demonstrate a superior breast and stomach cancer subtyping accuracy when integrating -omics in a GCN-based predictor. Likewise, Kesimoglu and Bozdag [64] used multiomics data combined with other raw features for GCN-based prediction of breast cancer subtypes. Interestingly, the derived subtypes had consistently significant survival differences that were mostly more significant than differences between the “ground truth” subtypes based on gene expression data, thus providing additional evidence in support of the superiority of multimodal analyses.

Fittingly, the latest work in this area combines information from multiple multimodal diagnostic disciplines in a single analysis scheme, taking advantage of GNN model representation flexibility and inter-domain transfer learning. For example, Furtney et al. [65] utilized radiographic images, genomics data, and other modalities to classify breast cancer subtypes via personalized breast cancer patient graphs.

In summary, we observe two trends: multilevel digital pathology data analysis and a broad, multimodal, approach to classification (that would ideally incorporate multilevel digital pathology, multiomics, and other features). While the former appears to be sufficiently mature, the latter is an emerging and promising trend; both significantly benefit from the ability of GNNs (often in cooperation with other DL modules) to combine different data types/modalities in a unified framework.

### 4.3. Granular Spatial Approaches (Including Transcriptomics and Proteomics)

Here, we are primarily concerned with the spatial single-cell analysis, and spatial heterogeneity, in tumor microenvironments. Early work in this area utilized “generalist” GNN-based approaches to spatially resolved gene expression analysis [66]. For example, Solorzano et al. [67] used GNNs for cell niche characterization in the glioma tissue. Subsequently, more complex, dedicated GNN models were developed to be applied in the cancer/oncology context. Zeng et al. [68] proposed a CNN–transformer–GNN architecture to capture spatially resolved RNA-seq expression from histology images, demonstrating high prediction accuracy for both gene expression and spatial region identification in cancer vs. normal datasets. Chang et al. [69] used graph autoencoder/GNN for spatially resolved transcriptomics in glioblastoma tissues, robustly classifying different regions. Qiu et al. [70] combined a variety of prognostic biomarkers (including molecular types) to model an “intratumor GNN” that captures spatial heterogeneity on different levels. The latter model’s prognostic performance proved superior on a retrospective breast cancer dataset. Likewise, Ding et al. [71] interwoven spatial profiles at different levels (WSI data, protein expression profiles, mutational profiles) to construct “spatially aware” multilevel GNN models from TCGA colon and rectum cancer data using a customized five-module GNN architecture. The latter models demonstrated high cross-level molecular profiles’ prediction accuracy on TCGA datasets. Wu et al. [72] used multiplex immunofluorescence imaging to show that a GNN leveraging spatial protein profiles adequately models tumor microenvironment via local subgraphs. Such subgraphs were found to be predictive for patient outcomes.

In summary, utilizing GNNs to “build the bridge” from cell-level spatial heterogeneity in tumor microenvironments to spatial region identification and cancer patient-level prediction tasks is a novel but highly promising research direction. We expect GNNs to play a crucial instrumental role in this area, as they are a seamless fit with multilevel spatial representations.

### 4.4. Cancer Drug Selection, Repurposing, and Profiling; Prediction of Cancer Drug Interactions and Combinations, Response, and Resistance

This broad area is especially amenable to GNN application due to the graph structures being the naturally commensurate representations for the chemical structures, drug–drug networks, and other multimodal networks incorporating diverse drug-relevant information. It is, therefore, not surprising that some of the earliest work in cancer/oncology GNNs was focused on graph models for drug and drug interaction representations. Cui et al. [73] adapted a generalist GCN to the task of drug repurposing against breast cancer, merging drug–drug networks with drug-exposure gene expression data. The resulting models outperformed both “classic” ML and standard DL approaches. In a reverse scenario, Gonzales et al. [74] used a GCN model to predict anticancer molecules within food (“superfoods”) based on a graph (human interactome) drug representation similarities to those of FDA-approved anticancer drugs, with the resulting models demonstrating both high prediction accuracy and interpretability. In parallel, Gao et al. [75] utilized multilevel (from atomic to molecule) drug structure graph representations to select candidate breast cancer drugs; thereby underscoring the two-pronged (molecular structure and drug-relevant networks) utility of GNN approaches.

Another prominent activity, complementing Section 4.1. above (therapy response prediction), is the prediction of a patient’s response to anticancer drug therapy, or a cancer cell line response to a drug. Zuo et al. [76] combined molecular structure graphs and gene features (expression, mutation) in a GNN–CNN model that showed superior performance on the benchmark Genomics of Drug Sensitivity in Cancer (GDSC) and Cancer Cell Line Encyclopedia (CCLE) datasets. Zhu et al. [77] added a different modality, protein–protein-interaction (PPI) networks (from the STRING database), and combined PPI and molecular graphs in a two-encoder GNN architecture for anticancer drug response prediction; likewise demonstrating performance advantages over the baseline non-GNN methods across different cancer cell line datasets. Liu et al. [78] added multiomics cancer cell line profiles to a GNN model, achieving performance improvements as well. Narrowing the focus to a specific group of drugs, Pu et al. [79] integrated genomics, biological networks, inhibitor profiling, and gene–disease associations in a unified GNN model to predict response to kinase inhibitors across various cancer tissues/cell lines. Similarly, Singha et al. [80] integrated multiple heterogeneous data in a GAT model for evaluating kinase inhibitors across different cancer cell lines. Emphasizing the interpretability of GNNs, Shin et al. [81] incorporated expert/domain knowledge (on biological pathways) in the multiple subgraphs–transformer model for anticancer drug response prediction, demonstrating improved performance on the GDSC datasets. Wang et al. [82] focused on the interpretability as well, applying a pruning mechanism to their multimodal drug response prediction GNN-based model. In general, it appears that adding additional heterogeneous information types to drug response prediction GNN models increases their generalization performance. It is serendipitous that GNNs are especially well-suited to such expansion.

An interesting variation on the theme was proposed by Peng et al. [83], wherein feature representations of drugs and cell lines are directly integrated in a heterogeneous network (instead of a bipartite graph). The latter model performed especially well on the GDSC and CCLE datasets. In parallel, Liu et al. [84] proposed a novel GNN architecture constructed around multiview graphs, with each input data type (various -omics, PPI) contributing a separate “view” to the multimodal drug response prediction. Automated optimization of GNN architectures in the cancer drug response prediction context is the latest trend in this research area, pointing to its relative maturity. Recently, Oloulade et al. [85] developed a framework for automated GNN hyperparameter/architecture optimization specifically tailored to each particular drug sensitivity dataset that consistently outperformed baseline methods from the first optimization epoch.

Moving on from single drugs to drug combinations, Wang et al. [86] used a GAT model to predict drug–drug synergy on cancer cells from the feature embedding of drug molecular structure and gene expression. Their model showed both high performance (+16 percent predictive precision over non-GNN methods on the AstraZeneca independent dataset) and interpretability. Notably, the latter led to gaining useful insights into the chemical substructure of anticancer drugs; yet again illustrating the added value of GNNs in contrast to “black box” methods. Bao et al. [87] also emphasized GNNs’ interpretability aiding in identifying molecular substructures contributing to drug synergy. An interesting additional aspect of this work was accounting for asymmetries in drug input, thus increasing predictive performance. Dong et al. [88] took this approach one step further, explicitly concentrating on identifying the mechanisms of synergy by dissecting the most salient molecular substructures revealed in their GAT model. Conversely, Ren et al. [89] constructed a GNN-based “biomedical knowledge graph” model with NLP (natural language processing) drug sequence semantics input to predict drug–drug interactions. The latter model showed high performance on cancer-related tasks.

In summary, GNNs’ ability to combine both molecular-structure-level and network-level data in interpretable models bodes well for significant further progress in this domain. Two particularly promising research directions are: (i) automated GNN hyperparameter/architecture optimization for each particular drug sensitivity dataset, and (ii) identification of molecular substructures most salient for anticancer drug synergism.

### 4.5. Synthetic Lethality Prediction

Synthetic lethality (SL) is a situation in which defects in two genes impair cell viability, but a defect in a single gene (of a pair) does not. If one gene is a cancer-specific defective gene, then targeting the other gene will lead to cancer cell death, while sparing non-cancerous, normal cells. Thus, in silico SL prediction emerged as one of the most effective methods for anticancer drug identification. Cai et al. [90], Wang et al. [91], and Lai et al. [92] pioneered the application of multimodal GCN to SL prediction and demonstrated superior performance on the human SL datasets compared to the non-graph-representation in silico SL prediction methods. Liu et al. [93] added features extracted from multiomics data to the GNN framework, thus expanding gene representation for SL prediction. Notably, the latter work exploited the interpretability of the graph representation to explain the SL mechanism. Likewise, Zhu et al. [94] focused predominantly on gene-related knowledge graph interpretability (without losing the predictive performance). Most recently, Fan et al. [95] developed a more complex, multiview GCN architecture, incorporating five biological modalities in a high-performance SL predictor.

In summary, SL prediction with gene graph representation is a relatively young but highly promising research area. We expect future research to concentrate on: (i) refinement of GNN architectures beyond “vanilla” GCNs, (ii) dissection of the SL mechanisms, enabled by the GNN’s interpretability, and (iii) integration of additional modalities in gene graph representations.

### 4.6. Prediction of ncRNA (miRNA, piRNA, lncRNA) and circRNA–Cancer Associations

Prediction of ncRNA–disease associations is a robust and well-established computational biology research field. GNNs can efficiently represent the interplay between ncRNA similarity networks and disease similarity networks. This potential was recognized early in the emergence of GNNs [96,97,98,99]. Subsequent and recent work in the context of cancer included using GNN models for miRNA–cancer association prediction [100,101,102,103,104,105,106], piRNA–cancer association prediction [107], lncRNA–cancer association prediction [108,109,110,111], and circRNA-cancer association prediction [112]. An interesting recent development is using multimodal GNNs to predict association not with disease but with anticancer drug resistance—for example, Liu et al. [113] incorporated disease-related information into the multimodal GNN predictor of circRNA–drug resistance associations, while Gao and Shang [114] used a GAT model for identifying lncRNA–drug resistance associations.

In summary, applying GNNs to dissect ncRNA–cancer associations is a mature field. We see future research progress as being largely incremental, with further architecture refinements and extensions in the multivariate directions (e.g., identifying ncRNA–cancer associations together with ncRNA–anticancer drug resistance associations, identifying ncRNA–multi-disease associations).

### 4.7. Other Research Directions, Activities, and Modalities

There is a variety of innovative and promising GNN-based cancer and oncology research situated outside of the above six categories (Section 4.1, Section 4.2, Section 4.3, Section 4.4, Section 4.5, Section 4.6). Some of the earliest work in the cancer–GNN junction aimed at the prediction of cancer driver genes with GCNs [115]. This was followed with a comprehensive study by Song et al. [116] developing a robust multimodal (36 features plus PPI) GAT-centered framework for the identification of driver genes across different cancers. Yang et al. [117] focused on a narrower problem of identifying a small number of genes for a cancer-specific tumor mutational burden estimation panel, essential for estimating the potential effectiveness of immune checkpoint inhibitor therapy. On the subject of immunotherapy, Wu et al. [118] developed a multimodal GAT-centered platform for neoantigen immunogenicity prediction. Combined with a comprehensive database of experimentally validated neoantigens, this platform provides a bridge to the clinical application of neoantigen-based cancer immunotherapy.

Chen et al. [119] used a GCN–SVM (support vector machine) architecture to combine disease similarity networks with metabolite similarity networks in order to identify ovarian-cancer-related metabolites. Fradkin et al. [120] developed a GAT model for molecule carcinogenicity prediction, demonstrating high generalization prediction accuracy. These two studies once again demonstrate the multilevel representation scope of GNN models, from the ontology networks down to molecular structures.

Several recent studies applied GNN representation and learning to radiotherapy optimization and planning. Kafaei et al. [121] developed a GNN/reinforcement learning model for simultaneous beam orientation and trajectory optimization of Cyberknife, achieving shorter treatment times without compromising the efficacy of radiotherapy. Shao et al. [122] used a GNN representation (from a single onboard X-ray projection) of a liver surface model that accurately translated, via real-time biomechanical modeling, to liver tumor localization; thereby optimizing image-guided radiotherapy. Subsequently, Shao et al. [123] incorporated surface imaging in the above framework. A clinical decision support system for response-adaptive radiotherapy developed by Niraula et al. [124] used GNNs to model inter-predictive-feature relationships and avoid nonphysical treatment response, demonstrating performance improvements on clinical decision-making.

In summary, there are still many hitherto unexplored (or explored to a limited degree, such as in the case of radiotherapy planning) areas of application of GNNs to cancer. Broadly speaking, if the input data/information can be naturally represented in a graph structure form, and if the dataset size/dimensionality suggests DL, investigators should consider GNNs. Even if only one data type or modality fits better with a graph representation, adding a GNN module to a complex DL architecture might improve both overall performance and interpretability. Alternatively, oftentimes features generated from non-graph modalities can best be integrated in a graph form. Higher interpretability and multilevel or multimodal representation are the crucial added value that GNNs contribute to the analysis pipeline.

## 5. Discussion

### 5.1. Pragmatic Considerations for GNN Deployment

The question of whether to use GNNs (as opposed, or in addition, to “vanilla” deep learning) in the predictive analysis and modeling of cancer- and oncology-research-related big data largely comes down to the data types and modalities. If one or more of the latter are more naturally represented in a graph structure form, then GNNs are indicated. Such data may include chemical structures, gene co-expression networks, PPI networks, drug-drug networks, spatially resolved imaging data, digital pathology data, patient networks in various clinical and epidemiological contexts, knowledge graphs, and multimodal biological networks in general. The actual modus operandi might be a GNN used for feature extraction followed by a DL predictor, or diffusion of information over a multimodal graph, or incorporation of a knowledge graph in the DL architecture. Numerous, increasingly sophisticated multicluster GNN-containing DL architectures are currently being developed to address diverse cancer and oncology research problems in a customized fashion.

There are three major advantages to GNNs, with two of them largely self-evident: intrinsic capability for multimodality (handling different data types in the same analysis framework) and interpretability (graph structures are more intuitive than layers and weights). The third advantage, higher predictive performance, is less immediately obvious, but has been amply demonstrated across the different tasks (Section 4.1, Section 4.2, Section 4.3, Section 4.4, Section 4.5, Section 4.6, Section 4.7), and can probably be at least partially attributed to less contextual information loss in the GNN/DL pipelines, and easier harmonization of different data types. It is important to remember that, although higher interpretability and more natural data structure representations are always desirable in and of themselves, the primary goal remains higher predictive accuracy—and it is gratifying to observe that GNN-centered architectures are at least as high-performing as more established baseline and state-of-the-art non-graph DL models.

The choice of GNNs vs. graphical models is less straightforward. Here, the two primary considerations should be the main activity (prediction vs. model selection/dissection, respectively) and data dimensionality. GNNs, and DL in general, achieve high predictive performance on large datasets, but their mechanistic and causal interpretability is still limited (even in the case of GNNs) in comparison with probabilistic graphical models. A big part of this is the ability of probabilistic graphical models, such as BNs, to propagate probabilistic inference, and to model perturbations in silico. On a fundamental level, this reflects the principal difference in connectivity representation: belief propagation in probabilistic graphical models vs. message parsing in GNNs. GNNs are more efficient learners when the graph structures (topologies) are largely preset, such as when the networks (chemical structures, gene co-expression networks, PPI networks, drug–drug networks, hard-coded knowledge graphs, etc.) are imported from other analyses. Of course, GNNs can also be used for the data-driven model (topology) selection, via edge-level tasks, just as graphical models can be used for node prediction and graph-level tasks, but these are not the primary motivations behind their respective applications.

To give a broad recommendation, if the features are well-defined, the datasets are not gigantic, and the primary activity is the mechanistic model selection with subsequent dissection/interpretation, graphical models might be a more natural choice. However, if the investigators are more interested in high predictive accuracy, some of the topologies are known or hard-coded (at least initially), and the data is big and features diffuse, the GNN/DL approach appears to be superior (and faster). That being said, the latest work in the field suggests a trend towards bridging the gap between graphical and causal models, on the one hand, and GNNs, on the other. For example, Li et al. [51] used GNNs to infer causative tumor features from CT data. More broadly, Vu and Thai [125] and Hua et al. [126] elaborated on the probabilistic explainability of GNNs and potential GNN–probabilistic graphical models synergies, with the ultimate goal being “probabilistic graphical models-enhanced GNNs” or, conversely, “GNN-enhanced probabilistic graphical models”.

### 5.2. Challenges and Future Directions

We see two major interrelated challenges to the broader acceptance and deployment of the GNN methodology in cancer and oncology research settings. First, the sheer novelty of the technique(s)—it is unclear if the potential performance benefits over “traditional” big data DL make it worthwhile to explore new and more complex architectures. To address this concern, in this review we have demonstrated that GNNs tend to outperform non-graph DL approaches across the board when the data types/modalities are amenable to the graph representation, with the added benefit of interpretability. However, this brings us to the second, more daunting, challenge: an absence of independent and comprehensive realistic cross-benchmarking studies for many, if not most, cancer- and oncology-related data analysis activities enumerated in Section 4.1, Section 4.2, Section 4.3, Section 4.4, Section 4.5, Section 4.6, Section 4.7. Having such studies, augmented with robust model evaluation metrics (beyond the standard AUC-ROC for classification tasks) is customary in the more mature fields in computational biology and medicine, ranging from phylogenetic analysis methods to gene regulatory network inference and tumor imaging segmentation, to name just a few. Conducting similar studies in the cancer and oncology research domain will go a long way toward the wider acceptance of GNNs. Our intuition is that GNNs will indeed prove superior overall, but this remains to be convincingly demonstrated to a broad audience. Such a demonstration should adopt and utilize more sophisticated model evaluation metrics, applicable to the graph and network structures. There is a wealth of appropriate well-established benchmark datasets and “ground truth” knowledge in the domains covered in Section 4.1, Section 4.2, Section 4.3, Section 4.4, Section 4.5, Section 4.6, Section 4.7, so we are optimistic that the comprehensive independent cross-benchmarking studies are forthcoming. They are sorely needed.

That being said, in our surveying of the field we have identified at least six sufficiently mature research directions (Section 4.1, Section 4.2, Section 4.3, Section 4.4, Section 4.5, Section 4.6). In our opinion, the most promising future methodological research directions for the next few years will be: (i) development of “boutique” GNN-containing DL architectures specifically tailored to various combinations of modalities and predictive tasks, (ii) automated optimization of said architectures and training regimes, (iii) direct incorporation of human expertise into prediction and decision pipelines, (iv) incorporation of additional modalities, on many levels, into multiscale graphs and models, and (v) extension to multivariate predictions. As far as actual cancer and oncology research tasks are concerned, we expect strong and growing research efforts in the areas of: (i) cancer classification and subtyping using digital pathology augmented by other modalities, (ii) dissection of spatial heterogeneity in tumor microenvironments with an eye towards patient-level predictions, (iii) identification of molecular sub-structures most salient for anticancer drug synergism and synthetic lethality prediction, (iv) real-time radiotherapy planning, and (v) multimodal prediction of immunotherapy response.

## 6. Conclusions

GNNs appear to be superior to non-graph DL approaches in many cancer and oncology research settings, particularly when the data is at least partially structured and multimodal, and when interpretability is desired. We anticipate that the future availability of independent and comprehensive cross-benchmarking studies will stimulate the broader acceptance of the GNN methodology in the field. From a different perspective, GNNs largely complement probabilistic graphical models, and we expect the increasing synergy between these two groups of models in the future. Cancer and oncology researchers and physician-scientists should consider GNNs as their principal secondary data analysis and predictive modeling tool if the data is big, multimodal, and one or more of the data types/modalities can be naturally represented as graph structures.

## Data Availability

Data are contained within the article.
